# Post-pandemic Work Motivation, Work Behavior and Psychic Structure in University Professors

**DOI:** 10.11621/pir.2024.0407

**Published:** 2024-12-15

**Authors:** Pablo Fernández-Arias, Álvaro Antón-Sancho, César J. Antona, Diego Vergara

**Affiliations:** a Catholic University of Ávila, Spain

**Keywords:** burnout, work experience, area of knowledge, emotional stability, COVID-19

## Abstract

**Background:**

Because of the pandemic, it has been suggested that motivation and job performance may have declined in various professional groups, a phenomenon known as *quiet quitting.* This study focuses on understanding this issue and its broader effect within the educational sector.

**Objective:**

This study analyses work motivation, work behavior, and psychic structure in a sample of university professors from Latin American and Caribbean countries.

**Design:**

A total of 612 professors from Argentina, Bolivia, Ecuador, El Salvador, Honduras, Panama, Paraguay and Peru participated in the study. Of the total sample, 379 (61.93%) of them were female. A subset of nine questions from the Bochum Inventory of Personality and Skills (BIP; Arribas et al., 2006) was used as the research instrument. The evaluation employed a cross-sectional multivariate factorial design.

**Results:**

The data revealed high overall scores and suggested a general profile of motivation, commitment and personal stability. Among the most salient results linked to years of teaching experience, statistically significant differences were observed between teachers at the beginning and end of their working careers compared to the scores obtained in the intermediate years of experience. The lowest scores derived from the majority of dependent variables were found among teachers with the least work experience. In contrast, teachers with more than 25 years of teaching experience presented the highest values in those constructs such as emotional stability and self-confidence. The area of subject matter which teachers taught (technical or humanistic) also significantly influenced the main variables evaluated.

**Conclusion:**

The evaluated university teachers did not present problems in terms of work motivation, work behavior and psychological structure. In other words, overall, they do not align with the phenomenon described as quiet quitting. However, the first years of teaching experience may be the most challenging for teachers to navigate, a factor that institutions may want to consider.

## Introduction

Since April 2021, more than 4 million workers have quit their jobs each month in the United States (Sahin & Tasci, 2022; Tessema et al., 2022). During 2022, the number of workers leaving their jobs voluntarily rose to 5.6 million, the highest figure since records began in 2001 (The Bureau of Labor Statistics, 2023). This phenomenon has also affected other Western countries (Kuzior et al., 2022). At the same time, a reduction in workers’ commitment, referred to a *quiet quitting,* has been suggested as a concurrent phenomenon. This does not involve quitting major work duties but reducing commitment to activities requiring extra time or attention to non-compulsory matters (Tessema et al., 2022). The main factors contributing to quiet quitting include: (a) the psychological impact of the COVID-19 pandemic (Brooks et al., 2020; Jiskrova et al., 2022); (b) a change in priorities with increased emphasis on health, well-being and family; (c) a perceived absence of appreciation from bosses, organizations and institutions. Indicative of the perceived increase of this phenomenon., an instrument has recently been validated in Greece to assess the prevalence of quiet quitting (Galanis et al., 2023).

Quiet quitting was not unique to any given field of work, but occurred in all sectors and professions, including academia. Traditionally, teachers are one of the groups most at risk of suffering from work-related stress and burnout syndrome (Anderman, 2020; Urbina-Garcia, 2020). Attrition and professional desertion among teachers have been very high, especially among young educators, as verified in a study conducted in 25 countries across the Americas, Europe, Asia and Oceania (OECD, 2005). Given the prevalence of this phenomenon in the teaching profession, the experience of teachers may be particularly relevant to study this trend. This variable has been linked to self-efficacy (Wolters & Daugherty, 2007), although the relationship is complex. For example, in a study evaluating 1430 Canadian professors Klassen and Chiu (2010) observed that, although years of experience were associated with professor self-efficacy, self-efficacy gradually increased up to 23 years of experience before it decreased toward the end of their careers. Younger and less experienced professors presented higher levels of emotional exhaustion and depersonalization relative to their older colleagues (Antoniou et al., 2006). For example, a study evaluating 251 Colombian professors (Ibáñez et al., 2012) found an inverse relationship between emotional exhaustion and the variables of age and time in the position (i.e., older and more experienced professors reported less emotionally exhaustion)Similarly, older professors reported less emotional exhaustion and greater job fulfillment than younger ones (Droogenbroeck et al., 2014), although the relationship between age and burnout remains complex and yields inconclusive results (Capone et al., 2019). The relationship between years of teaching experience and variables linked to burnout appears to be influenced by numerous contextual factors (Fiorilli et al., 2016). Moreover, years worked did not significantly affect the level of stress and burnout experienced by professors (Galanakis et al., 2020).

In teaching and research, the main fields of knowledge include the scientifictechnical area and the humanistic-social area, both equally valuable but clearly differentiated by their objects of study. These two branches of knowledge have been examined to determine whether university professors working in their respective fields of specialization exhibited significant differences in adapting to the new digital environments created by the pandemic (Antón-Sancho et al., 2022). The results suggested that several factors may be involved, among which the level of technological development of each country was significant. The Antón-Sancho et al. (2021) study which evaluated 219 university professors, found a lower self-assessment of personal and interpersonal competencies among teachers in the scientific-technical field compared to those in the humanistic-social area. It should be noted that, for teachers, the choice of a particular subject area is often vocational and provides meaning for their teaching and research tasks. Job engagement, defined as a psychological sense of accomplishment, positively affects employees (e.g., satisfaction, commitment and performance) and institutions (Wood et al., 2020). Conversely, a lack of meaningful or purposeful employment is among the primary reasons for job disengagement (Bailey et al., 2019). In this study we aimed to determine whether teaching experience and the previously mentioned subject matter specialization were related to work motivation, work behavior and the psychic structure of university professors; more specifically, , whether aspects of quiet quitting were observed in their work behavior.

The constructs defined above have been studied extensively. Regarding work motivation, research focus is placed on the teacher’s leadership, manifested through effective goal setting, and a capacity to offer support that includes feedback that is specific, positive and motivating (Ryan & Deci, 2020). Other notable teacher behaviors include the fostering of student autonomy and order, which increase intrinsic motivation (Guay et al., 2016), predict classroom performance (Aelterman et al., 2019), and lead to greater engagement and well-being at all stages of education (Ryan & Deci, 2020) and across cultures (Nolen, 2020). Furthermore, outcome orientation influences effort and persistence in teaching (Dornyei & Ushioda, 2011). Regarding work behavior, teachers have an immediate impact, often evident in each class, on the interest and motivation of their students (Tsai et al., 2008). Flexibility in the classroom is very important, as it is correlated with listening to students, reinforcing effort and suggesting possibilities when students are uncertain about how to continue. Finally, in regarding to psychic structure, teachers who have already satisfied their needs for autonomy, competence and social relationships will be more emotionally stable and promote the motivation, commitment, initiative and adjustment of their pupils. Thus, the tendency of professors to experience positive feelings about their work behavior mediates their enthusiasm and commitment to work (Andreychik, 2019; Schoeps et al., 2019). Professors’ emotional regulation has been found to be strongly associated with emotional intelligence (Fernández-Berrocal et al., 2017). The capacity to experiencing positive emotions may help professors prevent emotional burnout and increase job satisfaction (Wu et al., 2019). Conversely, professors who experience an elevated frequency of unpleasant emotions report a lack of motivation and increased emotional exhaustion (Abós et al., 2019). Additionally, the intensity of unpleasant emotions correlated with emotional exhaustion and burnout (Chang, 2013; Fiorilli et al., 2016). Improved perception, understanding, expression and emotional management enhance professors’ communication with students and colleagues, fostering the creation and maintenance of healthy relationships (Hopman et al., 2018; Mayer et al., 2016).

The aim of the current research is to analyze the phenomenon of quiet quitting among university professors in Latin American and the Caribbean, considering teaching experience and the subject matter areas of knowledge in which they teach (i.e., scientific-technical or humanistic-social). Furthermore, dependent variables related to work motivation, work behaviors and aspects of the psychic structure are considered. The specific objectives are as follows: (a) to determine the general profile of the sample in relation to the three dependent variables - *work motivation*, *work behavior* and *psychic structure*; (b) to examine whether teaching experience influences the aforementioned dependent variables; and (c) to assess if the area of subject matter knowledge affects self-assessments of the dependent variables across different age groups. Additionally, the study analyses the factor structure and reliability of the questionnaire used. In this sense, two more objectives were added: (d) to perform factor analysis of the questionnaire responses, and to ascertain the percentage of total variance explained; and (e) to elicit data reflecting the reliability of the questionnaire according to Cronbach’s alpha index.

The main explanatory variable is *teaching experience*, a polytomous nominal variable with six possible values (less than or equal to 5 years, between 6 and 10 years, between 11 and 15 years, between 16 and 20 years, between 21 and 25 years, and more than 25 years). The secondary explanatory variable is the *area of knowledge*, a dichotomous nominal variable derived from the International Standard Classification of Education (ISCED) established by the United Nations Educational, Scientific and Cultural Organization (UNESCO, 2011). The study defines the following categories: a) the scientific-technical area includes experimental sciences, natural sciences, health sciences, statistics, mathematics and physics, and b) the humanistic-social area, encompassing arts and humanities, geography, sociology, law, economics and business. The dependent variables evaluated include self-assessments in: (a) work motivation, (b) work behavior, and (c) psychic structure. The following hypothesis are tested: (a) teaching experience influences professors’ self-assessments of work motivation and work behavior and (b) the area of knowledge influences the dependent variables as teaching experience increases.

## Method

### Participants

There were 612 university professors in the study, of which 379 (61.93 %) were female. The distribution of participants from each country is shown below. Peru represented the largest number of participants, with 282 respondents reflecting 46.1% of the total, followed by Argentina with 177 participants (28.9%) and Ecuador with 102 (16.7%). El Salvador included 27 participants (4.4%), whereas Bolivia, Honduras, Panama and Paraguay were minimally represented, each with only 6 participants, representing 1.0% of the total.

### Questionnaire

A subset of nine questions from the Bochum Inventory of Personality and Skills (BIP; Arribas et al., 2006) was used as the research instrument. The original questionnaire demonstrates strong psychometric properties and has been specifically designed and adapted for use in Spanish-speaking countries. Previous studies have used subsets of the BIP and validated the corresponding instruments (Fernández-Arias et al., 2023). Our research team selected those items from the BIP that constituted a relevant and representative sample of the phenomenon of quiet quitting. We asked all teachers to assess their level of development and competence in three categories: (a) work motivation, (b) work behavior, (c) psychic structure. They assessed themselves on the following items: (1) initiative for change, (2) leadership, (3) results orientation (items related to work motivation), (4) conscientiousness, (5) flexibility, (6) action orientation (items related to work behavior), (7) work capacity, (8) emotional stability, and (9) self-confidence (items related to psychic structure). These items were measured on a Likert scale from 1 to 5 (1 indicating the lowest rating and 5 to the highest). We also provide a factor analysis and Pearson correlations to reinforce this validation.

### Procedure

Participants were selected using a non-probabilistic incidental sampling process. The evaluation was cross-sectional multivariate factorial. The sample consisted of university professors from Latin America and the Caribbean. Results were obtained in June 2023. Teachers’ countries lie within the same geographical area and, according to the Global Innovation Index (GII; WIPO, 2022), are homogeneous in terms of their level of innovation and digitization. The questions were sent to the target population through a GoogleForms^TM^ questionnaire.

### Data analysis

The validation of the instrument was performed by determining the latent factors that explain the variance through exploratory factor analysis. Pearson correlations between the ratings of the different constructs were computed to verify high correlations within the same group. Internal reliability was tested through Cronbach’s Alpha. After verifying that the responses were normally distributed, hypothesis were tested by analysis of variance (ANOVA) and multifactor analysis of variance (MANOVA). All statistical tests were performed with a significance level of 5%.

## Results

More professors in scientific-technical areas were represented (59.31%) compared to humanistic-social areas (4.69%; see *Table 1*). A homogeneous distribution of participants by areas of knowledge cannot be assumed (chi-square = 21.24, p-value < .0001).

**Table 1:** Distribution of participants by teaching experience and area of knowledge

Teaching experience	Scientific-technical (%)	Humanistic-Social (%)	N (%)
Less than or equal to 5 years	72.2	27.8	54 (8.8)
6 to 10 years	56.0	44.0	75 (12.3)
11 to 15 years	65.8	34.2	114 (18.6)
16 to 20 years	53.3	46.7	90 (14.7)
21 to 25 years	48.8	51.2	123 (2.1)
More than 25 years	63.5	36.5	156 (25.5)

*Note. The rows sum to 100% each.*

Both subject matter areas of knowledge are represented with at least 25% of the participants in each of the teaching experience ranges considered.

### Instrument validation

The factor analysis performed on the responses to the questionnaire showed three groups of constructs: *work motivation*, *work behavior* and *psychic structure* (*Table 2*).

**Table 2:** Factorial weights of the exploratory factor analysis

	Factor 1 Work motivation	Factor 2 Work behavior	Factor 3 Psychic structure
Initiative for change	.724		
Leadership	.739		
Results orientation	.772		
Concientiousness		.732	
Flexibility		.609	
Action orientation		.637	
Work capacity			.639
Emotional stability			.759
Self-confidence			.788

The grouping of the items analyzed into the three identified groups in the factor analysis defines a theoretical model that aligns with the BIP. Moreover, this theoretical model accounts for 68.1% of the total variance of the responses. The proportion of variance explained by the model which includes the three families of constructs obtained in the exploratory factor analysis is as follows: work motivation (.243), work behavior (.204) y psychic structure (.234). Finally, the Pearson correlation coefficients for the different constructs considered are consistently greater than .60 within each of the families defined by the theoretical model (*Table 3*). All the correlation coefficients presented are statistically significant.

**Table 3:** Pearson correlation coefficients between the different skills analyzed (all p-values are <.05)

	Initiative for change	Leadership	Results orientation	Conscientiousness	Flexibility	Action orientation	Work capacity	Emotional stability	Selfconfidence
Initiative for change	1								
Leadership	.67	1							
Results orientation	.65	.68	1						
Conscientiousness	.48	.49	.41	1					
Flexibility	.55	.44	.47	.65	1				
Action orientation	.49	.58	.54	.66	.68	1			
Work capacity	.46	.50	.44	.61	.59	.57	1		
Emotional stability	.40	.39	.36	.42	.57	.43	.64	1	
Self-confidence	.33	.49	.37	.51	.55	.55	.69	.70	1

The internal consistency was measured using the Cronbach’s Alphas, which are adequate, ensuring the internal reliability of the instrument. Their values are shown, in succession, for each construct: work motivation (.8575), work behavior (.8551) and psychic structure (.8634).

The assessments obtained are very high in three categories: work motivation (M = 4.20; SD = .86), work behavior (M = 4.41; SD = .71), and psychic structure (M = 4.42; SD= .75). The mean motivation towards work is significantly lower than the mean rating of work behavior, as the t-test for means highlights (t = 8.24, p-value < .0001) and the rating of psychic structure skills (t = –8.62, p-value < .0001). The Kolmogorov-Smirnov normality test indicates that the responses are not normally distributed (D = .24, p < .0001 for work motivation; D = .31, p < .0001 for work behavior; and D = .33, p < .0001 for psychic structure). However, the sample size endures the strength of the parametric tests used (mainly t-tests, ANOVA and MANOVA).

The ANOVA indicates that teaching experience significantly influences the mean self-assessments expressed for the three categories analyzed (note that all p-values in *Table 4* are less than .05). It can be assumed that: (a) professors with less than 5 years of teaching experience give lower ratings than the rest in the three constructs analyzed (paired p-values confirm that the differences between these professors and the rest are significant); and (b) for work motivation and work behavior, professors with more than 25 years of teaching experience give lower ratings than professors with between 16 and 25 years of experience (also in this case the paired p-values prove that the differences are significant). This latter observation would support the idea that, among university professors, there is a decrease in work motivation toward the end of their career. Therefore, results confirm the hypothesis that teaching experience significantly influences the assessed constructs.

**Table 4:** Mean responses according to the teaching experience and statistics of the ANOVA test for comparison of means.

	≤5 years	6–10 years	11–15 years	16–20 years	21–25 years	>25 years	F- statistic	p-value
Work motivation	3.80	4.20	4.09	4.37	4.36	4.19	12.10	< .0001*
Work behavior	4.15	4.48	4.40	4.46	4.48	4.39	4.26	.0008*
Psychic structure	4.33	4.35	4.46	4.41	4.33	4.56	6.27	< .0001*

The results concerning the influence of subject matter area of knowledge on the variance in evaluations made according to teaching experience are presented, differentiating each of the three constructs studied (*Table 5*).

**Table 5:** Work motivation, work behavior and psychic structure differentiated by teaching experience and area of knowledge

	≤5 years	6–10 years	11–15 years	16–20 years	21–25 years	>25 years
		Work motivation				
Scientific-technical	3.77	4.12	4.15	4.46	4.35	4.01
Humanistic-social	3.87	4.30	3.98	4.26	4.37	4.49
		Work behavior				
Scientific-technical	4.10	4.36	4.28	4.50	4.43	4.35
Humanistic-social	4.27	4.64	4.64	4.41	4.52	4.46
		Psychic structure				
Scientific-technical	4.36	4.40	4.40	4.58	4.27	4.51
Humanistic-social	4.27	4.27	4.56	4.21	4.38	4.65

The MANOVA test shows that the area of knowledge is the best explanatory factor for how mean perceptions vary with teaching experience. Moreover, the MANOVA test confirms that the area of knowledge has a significant influence (F = 8.19, p- value < .0001) on work motivation. Specifically, among professors in scientific-technical areas, there is a decrease in the ratings among professors with more than 25 years of experience (they follow the trend of the entire population). However, this decrease does not occur among professors in humanistic-social areas. There is also a significant correlation between the area of knowledge and the evaluations of work behavior (F = 3.64, p-value = .0028). Specifically, among professors in scientific-technical areas, there is a decrease in ratings after 25 years of experience. However, this decrease in ratings is observed among professors in humanistic-social areas after 15 years of experience (among them, the highest ratings occur between 6 and 15 years of teaching experience). Regarding psychic structure, growth is perceived after 25 years of experience in both areas of knowledge analyzed. However, in the scientific-technical area there is also a growth after 5 years of experience, while in the humanistic-social area this does not occur until 10 years of experience is reached. This difference between areas of knowledge is statistically significant (F = 5.84, p-value < .0001). Finally, the hypothesis regarding the evolution of self-report scores in relation to teaching experience, as it differs between professors in scientific-technical areas and those in humanistic-social areas, can be considered confirmed.

## Discussion

The study provides data derived from questionnaire responses of 612 university professors, representing a wide range of work experience, from novice professors to those with more than 25 years of experience. Their overall scores on the main dependent variables (work motivation, work behavior and psychic structure) were high and suggest a general profile of motivation, commitment and personal stability (the latter two factors being statistically higher than the first). A more detailed analysis of the data reveals significant differences between professors at the beginning and the end of their careers, in relation to the scores obtained in the intermediate years of experience. Therefore, professors with less than 5 years of experience present lower scores in the 3 variables mentioned above, while professors with more than 25 years of experience present a differential pattern. Regarding the latter (*Table 5*): (a) their work motivation differs notably according to their subject matter area of knowledge, (b) their work behavior exhibits intermediate values compared to the rest of the age groups, and (c) their self-perception of their psychic structure is very high.

The study includes a sample of equivalent professors who share the same profession and belong to a similar geographic and socioeconomic area. It was conducted using an evaluation questionnaire with adequate psychometric properties concerning the reliability and validity of its items and item groupings.

Work motivation and professional behavior have been studied for decades. They are influenced by individual factors (personality), structural factors (related to the job and the institution or specific work environment) and contextual factors (which include economic and social variables, as well as the effects of overcoming a pandemic). Among this amalgam of variables, the present study considers the impact of two other factors on the work motivation and job behavior of university professors: a) years of teaching experience and b) area of knowledge. These variables had a significant influence on motivation and job behavior and could be linked, at least partially, to the phenomenon of quiet quitting (Mahand & Caldwell, 2023).

### Teaching experience

Consistent with a variety of studies, teaching experience has been shown to be a variable that can affect the job performance of professors. For example, more experience has been associated with greater effectiveness (Wolters & Daugherty, 2007), although the relationship is observed to diminish after 23 years of experience (Klassen & Chiu, 2010). In addition to years of experience, other relevant variables appear to have an influence, including social support both inside and outside the institution (Fiorilli et al., 2016). While the literature tends to indicate younger professors with less work experience exhibit differences in their work motivation in relation to their more senior peers (Antoniou et al., 2006; Capone et al., 2019; Droogenbroeck et al., 2014; Ibáñez et al., 2012), not all studies observe differential effects between this variable (Galanakis et al., 2020). In the present study, it was observed that teachers with less than 5 years of experience presented statistically significant differences compared to the rest, revealing a pattern of lower work motivation and proactivity compared to their older colleagues (Table 4). Conversely , after the age of 25, a decrease in these factors was also observed. Professors with fewer years in the profession basically perform the same functions (teaching, research and management) without the accumulation of knowledge and experience acquired later in their careers. Additionally, younger subjects may not yet have as realistic a perception of their career as their more experienced colleagues. Consequently, they may not utilize the most suitable coping strategies (Antoniou et al., 2006) and may be very demanding of their families, especially the female subgroup.

Professors with more than 25 years of professional experience presented statistically lower scores in work motivation and work behavior compared to teachers with experience between 16 and 25 years (Table 4). The world of work is constantly changing, and technological development has experienced exponential growth. Therefore, more experienced workers may experience a certain obsolescence and greater stress in relation to the adoption of new technologies. Caution might suggest that the accumulation of stressful life events could be greater in more experienced workers, potentially leading to a change in priorities. Yet, the scores of the group of teachers with more than 25 years of experience are higher in psychic structure than those of other age groups (Table 4). These results are consistent with those suggesting that younger teachers suffer greater emotional exhaustion and experience less commitment to their profession than their more experienced colleagues (Antoniou et al., 2006). This observation may be relevant for universities.

#### Knowledge areas

The professors’ subject matter area of knowledge significantly influences the three dependent variables (Table 5). In relation to work motivation, understood as achievement motivation, involves performing to the best of one’s ability. It is acquired during childhood and adolescence (strongly influenced by the family environment), remains relatively stable throughout the life cycle and is strongly linked to personality (Morán & Menezes, 2016). The group of teachers with less than 5 years of experience had the lowest scores pertaining this variable; the fewer the years of experience, the lower the leadership capacity and initiative to make changes, possibly because they have less influence within the educational institution. Subsequently, it rises in the two groups in the intermediate phases and differs notably between them in the later stages.

In terms of area of knowledge, in general, the humanistic-social area tends to show higher levels in all three variables, especially in professors with more than 25 years of experience. Work motivation in the scientific-technical area reaches its maximum in the 16-20 years age group, while in the humanistic-social area it is highest in the group with more than 25 years. Work behavior shows a similar trend, with generally higher scores in the humanistic-social area. The psychic structure shows more pronounced fluctuations, with the scientific-technical area peaking in the 16-20 age group, while the humanistic-social area shows its highest score in the group with more than 25 years of experience.

Conversely, if a job is perceived as meaningful and has some personal significance, it may affect achievement motivation and drive job improvement (Bailey et al., 2019). A job’s meaning and significance as perceived by university professors may be linked to their general performance , but it may also be affected by the subject matter taught and the topics they research. That is, to the extent that the area of knowledge is related to a vocational choice, it could be thought that there is greater motivation and pleasure in work performance. Thus, the results of this study indicate that professors in the humanities with more than 25 years of experience presented the greatest difference in work motivation comparted to their colleagues in the scientific-technical area (Table 5). In this sense, in the area of scientific and technical knowledge, these scores can be related to quitting, but this is not the case in the area of humanities where the subgroup of professors over 25 years of age presented higher value than the rest of the age groups.

Of the three dependent variables, work motivation exhibited the greatest variability. In the initial stages of professors’ careers, there may be a greater risk of job abandonment. Additionally, from an institutional point of view, the evaluation and analysis of each employee may be considered in order to identify those individuals who may present greater difficulties.

The area of knowledge also has a significant influence on work behavior. In this variable, the group of teachers with less than 5 years of experience presented the lowest scores (Table 5). The development of skills and competencies is acquired over time, and, early in a career, these tend to be lower. This idea is consistent with the view that difficulties at the beginning of teachers’ careers may be related to adaptation to the profession and may not necessarily pose a long-term problem (van Dick & Wagner, 2001). The data from the present study indicates that this is the case in the first half of the work cycle; however, the perception of one’s own work behavior decreases after 25 years of experience, with this decline being even more pronounced in the scientific-technical field (Table 5). In this sense, attention should be given to both the beginning and end of the work cycle.

Work motivation and work behavior vary significantly according to age group and area of knowledge, presenting differentiated profiles in the three dependent variables (Table 5). The profiles of the scores for workers in the scientific-technical knowledge area are very similar across the dependent variables including work motivation and work behavior, suggesting both are closely linked. For work behavior, teachers in the humanities with 6 to 15 years of experience rate themselves with the highest scores and, thereafter, scores fluctuate. As previously indicated, professors indicate their work motivation increases progressively after 15 years of experience.

In the realm of psychic structure, internal factors predominate and, consistent with personality trait theories, they are likely to remains stable throughout the life cycle. In the case of teachers with less than 5 years of work experience, scores in both the scientific-technical and humanities branches are low and suggest moderate self-confidence and emotional stability. Professors with more than 25 years of experience, particularly those in the humanistic area of knowledge, exhibit exceptionally high values in the psychic structure variable. The progression of scores in the two areas of knowledge to which the teachers belong is not linear, rather they are low at the beginning of the work cycle and significantly higher at its conclusion. One of their main constructs is emotional stability, which indicates that may exhibit a high degree of affective (Fernández-Berrocal et al., 2017; Wu et al., 2019) and motivational balance (Andreychik, 2019; Schoeps et al., 2019), even if this does not correspond to sustaining the workload they managed earlier in their career. Moreover, greater emotional stability may mitigate symptoms such as emotional exhaustion (Chang, 2013; Fiorilli et al., 2016). The accumulation of experience may provide professors with a broader perspective as their years of work experience increase. However, personal factors also play a critical role, as learning from experiences depends not only on having undergone them, but also on the ability to understand and interpret them adaptively (Hopman et al., 2018; Mayer et al., 2016). The data suggest the development of an internal locus of control at the latter stages of a career, potentially due to the prolonged trajectory professional trajectory that provides opportunities for significant personal learning and growth. Although the beginning and end of a career present a similar pattern, differences are observed in the intermediate stages as scores present contrasting results (Table 5).

Regarding the objectives linked to the subset of the 9 selected items of the BIP, it is significant that: (a) the three factors observed align with those of the original instrument and account for almost 70% of the total variance measured, and (b) the internal reliability of this subset is high.

## Conclusion

Among the main results, it is worth noting that:

- The sample exhibits high scores in work motivation, work behavior and psychic structure.
- Professors with less than 5 years of experience exhibit statistically significant differences characterized by lower motivation and proactivity compared to their more experienced colleagues. The finding underscores the importance of addressing the risk of early career attrition within educational institutions.
- Professors with over 25 years of experience, particularly those in the humanities, achieved higher scores in psychic structure, encompassing factors such as emotional stability and self-confidence.
- The subject matter area of knowledge significantly influences motivation levels. Moreover, humanities professors with over 25 years of experience present the highest levels of work motivation compared to their colleagues in the scientific-technical fields.
- Science professors scored significantly lower than their colleagues in the humanities on work behavior.

Recommendation for future research include an increased personalization of the assessment instrument, particularly among age categories where scores across dependent variables have been lower. Factors that may influence work behavior, such as gender (a variable commonly linked to burnout), marital status, number of children or dependents, should be considered. Additionally, evaluating factors such as the perception of job security and the possible presence of other relevant stressful life events would provide valuable insights.

The primary sources of work stress could also be evaluated to determine whether they stem from interaction with students, superiors or peers, feeling overloaded with teaching changes and/or their research work, lack of continuous training, and other factors. Additionally, the profile of workers with more than 25 years of experience could be further analyzed, as this group is the most numerous, and potentially categorized into more significant segments according to years of experience. Finally, it would be valuable to assess the different teaching modalities (classroom, blended or online) to examine their impact on work-related stress and performance.

## Limitations

Among the limitations of the study is that the cross-sectional assessment could be complemented by a longitudinal assessment to mitigate threats related to the cohort effect such as the impact of cultural, technological, educational changes over time. With the conclusion of the pandemic, a reassessment of the results is appropriate. Another aspect to consider is the number of professors in some of the countries evaluated varied considerably, with some countries under-represented. Finally, the cultural and economic idiosyncrasies of the study’s context should be taken into account, as they may have implications for the generalizability of the findings to other geographical areas.
